# Iron Oxide Magnetic Nanoparticles with a Shell Made from Nanosilver—Synthesis Methodology and Characterization of Physicochemical and Biological Properties

**DOI:** 10.3390/ma15124050

**Published:** 2022-06-07

**Authors:** Magdalena Kędzierska, Anna Drabczyk, Mateusz Jamroży, Sonia Kudłacik-Kramarczyk, Magdalena Głąb, Piotr Potemski, Bożena Tyliszczak

**Affiliations:** 1 Department of Chemotherapy, Medical University of Lodz, Multidisciplinary M. Copernicus Voivodeship Center for Oncology and Traumatology (WWCOiT), 90-001 Lodz, Poland; magdalena.kedzierska@umed.lodz.pl (M.K.); piotr.potemski@umed.lodz.pl (P.P.); 2 Department of Materials Engineering, Faculty of Materials Engineering and Physics, Cracow University of Technology, 37 Jana Pawła II Av., 31-864 Krakow, Poland; magdalena.glab@doktorant.pk.edu.pl (M.G.); bozena.tyliszczak@pk.edu.pl (B.T.); 3 Faculty of Materials Engineering and Physics, Cracow University of Technology, 37 Jana Pawła II Av., 31-864 Krakow, Poland

**Keywords:** iron oxide magnetic nanoparticles, silver nanoparticles, core–shell nanostructures, ultrasound-assisted agglomerate crushing, arabic gum, colloidal stability, pro-inflammatory activity

## Abstract

The interest in magnetic nanoparticles is constantly growing, which is due to their unique properties, of which the most useful is the possibility of directing their movement via an external magnetic field. Thus, applications may be found for them as carriers in targeted drug delivery. These nanomaterials usually form a core in a core–shell structure, and a shell may be formed via various compounds. Here, nanosilver-shelled iron oxide magnetic nanoparticles were developed. Various reaction media and various Arabic gum (stabilizer) solution concentrations were investigated to verify those that were most beneficial one in limiting their agglomeration as much as possible. The essential oil of lavender was proposed as a component of such a medium; it was used alone or in combination with distilled water as a solvent of the stabilizer. The particle size was characterized by dynamic light scattering (DLS), the chemical structure was characterized via FT-IR spectroscopy, the crystallinity was characterized by X-ray diffraction (XRD), and the surface morphology and elemental composition were verified via the SEM-EDS technique. Moreover, UV-Vis spectrophotometry was used to verify the presence of the shell made of nanosilver. Importantly, the particles’ pro-inflammatory activity and cytotoxicity towards L929 murine fibroblasts were also characterized. It was demonstrated that a 3% stabilizer solution provided a preparation of Fe_3_O_4_@Ag particles, but its stabilizing effect was not sufficient, as a suspension with micrometric particles was obtained; thus it was necessary to apply 4 h of sonication for their crushing. Next, the oil/water reaction medium was verified as beneficial in terms of nanoparticle formation. In such reaction conditions, the formation of particle agglomerates was strongly limited, and after 15 min of sonication a suspension containing only nanoparticles was obtained. The presence of a nanosilver shell was confirmed spectrophotometrically via XRD and SEM-EDS techniques. Importantly, the developed nanomaterials showed no cytotoxicity towards murine fibroblasts and no pro-inflammatory activity.

## 1. Introduction

“There’s plenty of room at the bottom”—this sentence stated by Richard Feynman drew the attention of scientists to the miniaturization of the surrounding world, i.e., the possibilities and perspectives related to this phenomenon. The considerations of this scientist initiated studies on nanomaterials [1]. According to the definition, the term “nanomaterial” is defined as a material or a structure with at least one dimension in the range of 1–100 nm [2]. In turn, the science that deals with the fabrication of various structures, materials, and devices with the size of atoms or groups of atoms is nanotechnology. The most general division of methods for the preparation of nanomaterials is based on the “bottom-up” and “top-down” approaches [3]. The “top-down” method consists of the grinding of a macroscopic object until the material is nanometric in size, which may be achieved by dividing the starting material in a controlled manner so that at least one of its dimensions is within the range of 1–100 nm [4]. On the other hand, the procedure on which “bottom-up” methods are based is the opposite of that of “top-down” methods, i.e., instead of the fragmentation of macroscopic materials, these methods are based on building nanosized objects from smaller ones [5]. By adequately controlling the parameters of both of these approaches, it is possible to obtain objects of nanometric dimensions with the desired properties [6].

An interesting group of nanomaterials is that of magnetic nanoparticles. Their most unique feature is their reaction to applied magnetic fields, and this significantly increases the application potential of such nanostructures [7,8,9]. These nanomaterials show potential for use, e.g., for biomedical purposes, including targeted drug delivery, imaging, or biodetection [10,11]. Particularly interesting are the structures of core–shell-type nanomaterials. These are biphasic structures with an inner core and an outer shell made from different materials [12]. One of the types of such structures is one with a magnetic core, while the shell can be prepared using both inorganic and organic components [13,14]. For example, Chen et al. proposed Fe_3_O_4_@TiO_2_ core–shell structures with immobilized IgG, which were designed to inhibit the bacteria cell growth [15]. Next, Purushotham et al. presented core–shell materials consisting of a core made from γ- Fe_2_O_3_ iron oxide magnetic nanoparticles, while isopropylacrylamide was applied as a shell. This system was loaded with doxorubicin and developed for in vitro release of this active substance [16]. On the other hand, Liao et al. demonstrated the synthesis and characterization of iron oxide magnetic nanoparticles functionalized with alginate. These materials were subsequently linked to a specific ligand and used in hyperthermia therapy, which is applied in the treatment of cancer [17]. In turn, Zhang et al. proposed phosphatidylcholine as a shell of iron oxide nanoparticles, and these structures were designed for both magnetic resonance imaging and hyperthermic applications [18].

Nonetheless, one of the obstacles to the synthesis and use of magnetic nanoparticles is their high tendency to agglomerate. Such a phenomenon is caused by interactions between the hydrophobic surfaces of these nanomaterials. As a result, they form large agglomerates in which additional magnetic attractions occur between individual nanoparticles. In order to limit this phenomenon, the surface of the nanoparticles may be modified with select compounds of a synthetic or natural origin. In addition, new methods for their synthesis are being developed by selecting the reagents and the reaction conditions so as to maximize the colloidal stability of the obtained nanoparticle suspensions. Moreover, various stabilizers may be used to limit the interactions between the nanoparticles. The role of such stabilizing agents may be played, e.g., by Arabic gum. This is a non-toxic, hydrophilic biopolymer of plant origin. The main components of this substance are the calcium, potassium, and magnesium salts of arabinic acid (referred to as arabine). Its branched structure forms a coating on the surface of these nanomaterials, thus limiting their agglomeration. Furthermore, due to its chemical structure, i.e., the presence of charged functional groups, Arabic gum may be physically absorbed on the surfaces of nanoparticles. As a result, electrostatic interactions occur between the nanoparticles with such modified surfaces, preventing them from aggregating into agglomerates [19,20,21].

The main purpose of this work was to develop a synthesis methodology for iron oxide magnetic nanoparticles with a shell made from silver nanoparticles. In the first step of the research, the impacts of the concentration of the stabilizing agent applied (Arabic gum) and the type of the reaction medium used on the colloidal stability of the obtained magnetic nanoparticle suspensions were verified. The novelty of these investigations was the use of an essential oil as a component of the reaction medium. This was to create conditions for reducing the nanoparticle agglomeration. The size of the particles obtained was determined by dynamic light scattering (DLS), while sonication was used to break up the nanoparticle agglomerates. Next, the chemical structure of the particles was characterized by Fourier transform infrared (FT-IR) spectroscopy, their surface morphology and elemental composition were evaluated with scanning electron microscopy–energy-dispersive X-ray spectroscopy, and their crystallinity was analyzed via X-ray diffraction (XRD). Importantly, the pro-inflammatory activity of the particles obtained and their cytotoxicity towards L929 murine fibroblasts were also assessed.

## 2. Materials and Methods

### 2.1. Materials

Silver nitrate, which acted as a source of silver ions, and Arabic gum (yellowish powder, stabilizing agent) were purchased from Avantor Performance Materials Poland S.A. (Gliwice, Poland). In turn, hydroxylamine hydrochloride (98%, reducing agent) was bought from Sigma Aldrich (St. Louis, MO, USA). The plant essential oil (lavender) applied to form a reaction environment during the synthesis of the Fe_3_O_4_@Ag nanoparticles was purchased from AromaLab (Warsaw, Poland).

### 2.2. The Synthesis Methodology for Fe_3_O_4_@Ag Nanoparticles

Synthesis was performed using Fe_3_O_4_ magnetic nanoparticles that were previously obtained via Massart synthesis. This procedure was described in detail by Kędzierska et al. [22]. In order to prepare Fe_3_O_4_@Ag nanoparticles, 7.50 mL of an iron oxide nanoparticle suspension was added to an aqueous Arabic gum solution; the solution of this stabilizer was prepared with various concentrations, i.e., 1.0% and 3.0%. The mixture was heated to 80 °C. After reaching this temperature, 0.2 M sodium nitrate (3.75 mL), which acted as a source of silver ions (Ag^+^), and 0.1 M hydroxylamine hydrochloride (1.50 mL), which played the role of a reducing agent (to reduce Ag^+^ ions into Ag^0^), were added. This reduction was conducted for 1 h, and the temperature of 80 °C was maintained during the whole process. Moreover, Fe_3_O_4_@Ag nanoparticle synthesis was performed with constant stirring and in an inert gas (argon) atmosphere.

Importantly, the synthesis of Fe_3_O_4_@Ag nanoparticles was carried out using three different reaction environments, namely:

- aqueous solution of Arabic gum (water environment);
- mixture of the aqueous solution of Arabic gum and essential oil in a 1.4:1.0 volume ratio (oil/water (O/W) environment);
- solution of Arabic gum in the essential oil.

Various reaction environments and various stabilizing solution concentrations were used to verify the most favorable conditions for the preparation of a stable suspension of Fe_3_O_4_ nanoparticles.

### 2.3. Analysis of the Particle Size via Dynamic Light Scattering (DLS)

This research was focused on developing a synthesis methodology that would lead to the preparation of nanosized particles. Thus, it was essential to verify the size of the particles obtained. For this purpose, dynamic light scattering (DLS method) was used; the measurements were performed at ambient temperature and by using glass cuvettes. The study was carried out on a Zetasizer Nano ZS Malvern (Malvern Panalytical Ltd., Malvern, UK). Importantly, during each measurement, the polydispersity index (PI) was also verified.

In the case of agglomerates of particles, a sonication process was used for their disintegration. For this purpose, an Omni Sonic Ruptor ultrasound homogenizer (pulsation: 50%, power: 40%) was used.

### 2.4. Characterization of the Optical Properties of the Particles via UV-Vis Spectrophotometry

The formation of a nanosilver shell on the surface of the iron oxide magnetic nanoparticles was verified by using UV-Vis spectrophotometry. This part of the research was conducted by means of a ThermoScientific Evolution 220 UV-Vis spectrometer. The measurement was performed at room temperature within the wavelength range of 300–700 nm.

### 2.5. Analysis of the Chemical Structure of the Particles via FT-IR Spectroscopy

The presence of characteristic groups in the structure of the developed materials was determined using Fourier transform infrared (FT-IR) spectroscopy. For this purpose, a Thermo Scientific Nicolet iS5 FT-IR spectrophotometer (Thermo Fisher Scientific, Waltham, MA, USA) equipped with an iD7 ATR was used. FT-IR spectra were recorded within the 4000–400 cm^−1^ wavelength range at room temperature.

### 2.6. Evaluation of the Crystallinity of the Particles Obtained via X-ray Diffraction (XRD)

The particles’ crystallinity was characterized via the X-ray diffraction (XRD) technique. The analysis was carried out using a Bruker D2 Phaser diffractometer. The measurements were conducted at room temperature, in the reflection mode (kCu = 1.54 Å), and within the measurement range of 20–140°.

### 2.7. Analysis of the Surface Morphology Supported by the Identification of the Elemental Composition of the Particles via the SEM-EDS Technique

The application of a scanning electron microscope with an EDS detector (voltage: 10 kV) allowed the characterization the particles’ surface morphology. Importantly, the elemental composition of the obtained materials was also verified. The analysis was performed with a Helios NanoLab H50HP FEI (FEI, Hillsboro, OR, USA) microscope. Before the study, the samples of particle suspensions were lyophilized, and the powder obtained was sputtered with carbon.

### 2.8. The Particle Surface Morphology Analysis Using Transmission Electron Microscopy (TEM)

The particle surface morphology was analyzed using transmission electron microscopy. For this purpose, a JEOL JEM1200 (JEOL USA Inc., Peabody, MA, USA) transmission electron microscope was used. TEM images were recorded using an accelerating tension of 120 kV.

### 2.9. Biological Investigations

#### 2.9.1. Evaluation of the Cytotoxicity of the Particles via an MTT Reduction Assay

An important aspect of the research was the evaluation of the impact of the developed materials on selected cell lines. For this purpose, an MTT reduction assay was conducted. This involved determining the viability of the cells after 24 h of incubation with the tested materials. This, in turn, proceeded by means of verifying their metabolic activity. Properly functioning cells secrete numerous enzymes, including mitochondrial dehydrogenase. This substance converts the 3-(4,5-dimethylthiazol-2-yl)-2,5-diphenyl tetrazolium bromide (also known as MTT reagent) into insoluble formazan, which is then dissolved in a selected solvent (e.g., isopropanol or DMSO), and its amount (and concentration) may be verified via UV-Vis spectrophotometry. Thus, introducing the MTT reagent into the tested cell lines treated with the analyzed materials, followed by further spectrophotometric analysis of the formazan solution that formed, allowed the determination of the number of living cells. This part of the study was performed using L929 murine fibroblasts. The whole procedure of the MTT reduction assay and the cultures of the tested cell lines were described in detail in [23].

#### 2.9.2. Studies on the Pro-Inflammatory Activity of the Particles

Another biological investigation involved determining the pro-inflammatory activity of the particles. This part of the study was performed by using THP1XBlueTM cells (InvivoGen, San Diego, CA, USA), i.e., a monocytic macrophage cell line that was genetically modified. As a result of the cell response to the presence of pathogens or stress, the germline alkaline phosphatase (SEAP) was released by the cells into the analyzed medium. This substance, in turn, reacted with a QuantiBlueTM reagent to form a complex whose presence (and, thus, concentration) could be verified via UV-Vis spectrophotometry. The whole procedure of this study was previously described in [24].

## 3. Results and Discussion

### 3.1. Selection of the Stabilizer Concentration of the Particles’ Suspension Supported by the DLS and UV-Vis Analyses

**Figure 1:**
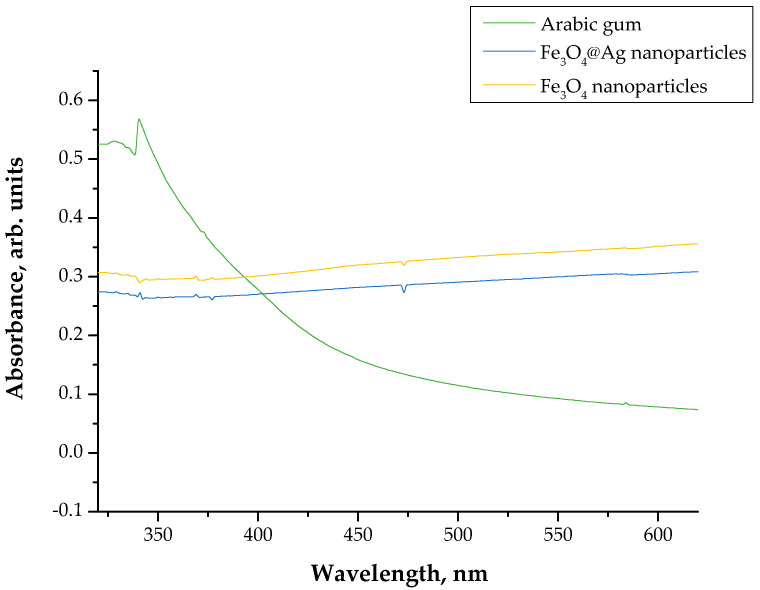
UV-Vis spectrum of the suspension obtained after the synthesis of Fe_3_O_4_@Ag particles using the 1% Arabic gum solution.

In Figure 1, the UV-Vis spectrum of the obtained Fe_3_O_4_@Ag nanoparticles is presented in comparison with the UV-Vis spectra of Fe_3_O_4_ nanoparticles and Arabic gum.

**Figure 2:**
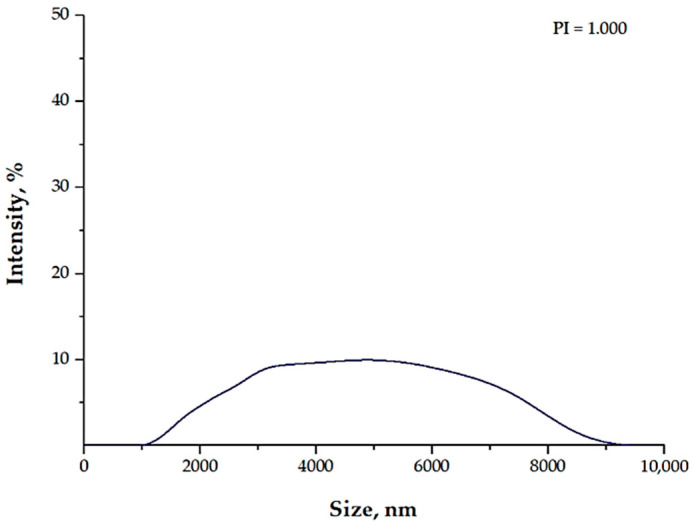
The size analysis of the particles in the suspension obtained as a result of the synthesis performed using the 1% Arabic gum solution.

The absorption band characteristic for silver nanoparticles is within the wavelength range of 380–450 nm [25]. In the UV-Vis spectrum obtained here, the presence of the absorption band within this range was not observed. Thus, it may be concluded that the nanosilver shell did not form on the surface of the magnetic nanoparticles. This proves that the 1% Arabic gum solution did not limit the agglomeration of iron oxide nanoparticles sufficiently and in a manner enabling the formation of a shell made from silver nanoparticles on the surface of Fe_3_O_4_ nanoparticles. In Figure 2, the results of the particle size analysis are presented.

The results of DLS analysis confirmed the effectiveness of the use of the stabilizing agent in the form of the 1% Arabic gum solution. Similar conclusions were drawn based on the UV-Vis spectrophotometry. In the analyzed suspension, the presence of particles with a micrometric size, i.e., within the range of 1000–9000 nm, was reported. This proved the low colloidal stability of the analyzed suspension, resulting in the formation of agglomerates of the magnetic nanoparticles. As a result, the formation of Fe_3_O_4_@Ag nanoparticles was strongly limited.

**Figure 3:**
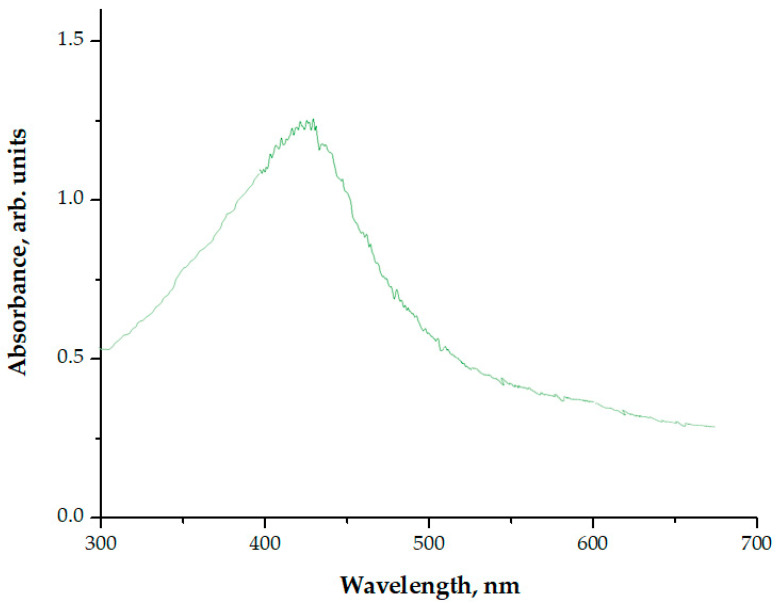
UV-Vis spectrum of the suspension obtained after the synthesis of Fe_3_O_4_@Ag particles using the 3% arabic gum solution.

In the next part of the research, the synthesis of nanosilver-shelled magnetic nanoparticles was conducted using a 3% Arabic gum solution as a stabilizer. In Figure 3, the results of the UV-Vis spectrophotometric analysis of the nanoparticle suspension obtained as a result of the reaction are presented.

**Figure 4:**
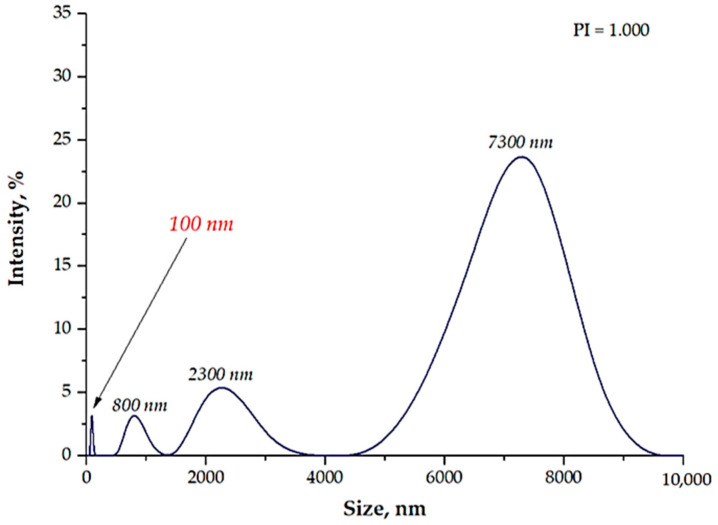
The size analysis of the particles in the suspension obtained as a result of the synthesis performed using the 3% arabic gum solution.

An absorption band with the maximum absorbance at a wavelength of approximately 430 nm was observed in the obtained UV-Vis spectrum. This value was within the absorption range characteristic of nanosilver. Thus, it may be concluded that, as a result of the reaction performed with the use of the 3% Arabic gum solution, magnetic nanoparticles with a shell made of nanosilver (Fe_3_O_4_@Ag) were synthesized. This proved the effectiveness of the applied stabilizing agent solution. Next, the size of the particles obtained was verified through a DLS analysis. The results are shown in Figure 4.

Based on the results of the DLS analysis, it was stated that the tested suspension showed a multimodal particle size distribution. The presence of particles with a size of approximately 800 nm (intensity of approximately 2.5%), approximately 2300 nm (intensity of approximately 5%), and 7300 nm (intensity of 25%) was shown. Furthermore, it should be emphasized that, in the suspension, particles with a nanometric size could also be observed. Thus, it may be stated that the use of Arabic gum in the form of a 3% aqueous solution as a stabilizing agent allowed us to obtain nanoparticles.

Arabic gum (also known as acacia gum) is a branched-chain hydrocolloid consisting of an arabino–galactan protein complex with magnesium, calcium, and potassium. It shows biocompatibility and biodegradability. Molecules of this biopolymer covered the surface of the Fe_3_O_4_ magnetic nanoparticles. As a result, the interactions between the nanoparticles were limited due to the so-called steric hindrance. The long polymer chains of the polysaccharides of Arabic gum (to be more precise, carboxylic acid salts) significantly reduced interactions that might lead to the formation of agglomerates. This phenomenon is defined as a steric stabilization. Furthermore, even a biopolymer that is not absorbed on the surface of nanoparticles, but only exists in the reaction medium in a dispersed form, limits the interactions of nanoparticles. Such a stabilization is defined as a depleted stabilization [26].

Nevertheless, for this to happen, it is necessary to use an appropriate concentration of the stabilizing agent. As was proven in the course of the study, the use of Arabic gum in the form of a 1% aqueous solution did not provide the expected results. In turn, the 3% aqueous solution of this biopolymer constituted an effective stabilizer and allowed for the preparation of particles with a nanometric size.

**Figure 5:**
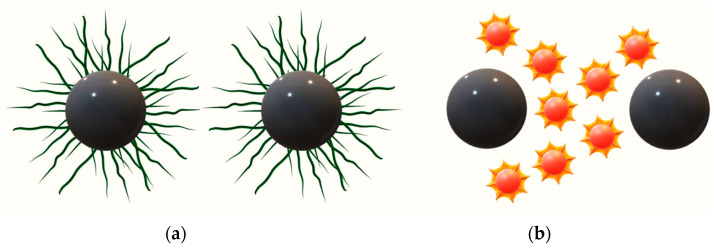
Types of stabilization of particles, i.e., steric (**a**) and depleted (**b**).

In Figure 5, the types of stabilization of nanoparticles are schematically presented.

**Figure 6:**
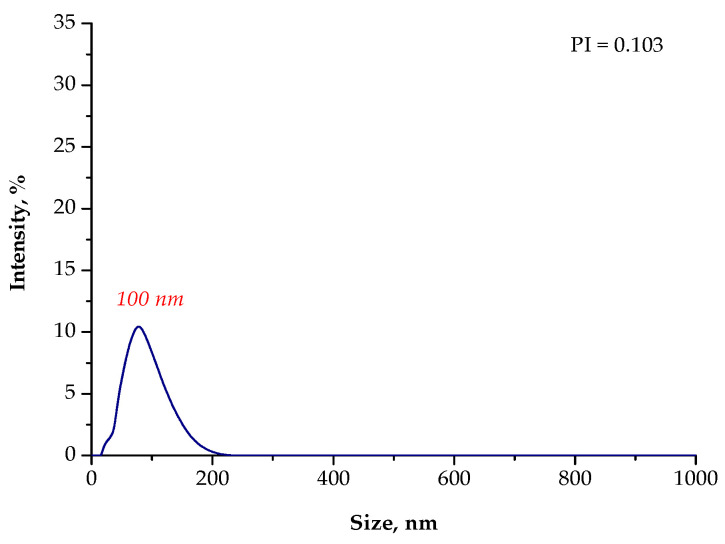
The size analysis of the particles in the suspension obtained as a result of the synthesis performed using the 3% Arabic gum solution after 4 h of sonication.

However, the stabilizing effect of Arabic gum was not sufficient. The particle suspension obtained was polydisperse and contained both micro- and nanoparticles. Thus, in order to crush the nanoparticle agglomerates, a sonication process based on the use of ultrasonic energy was performed. After the ultrasound-assisted agglomerate crushing, the size of the particles was verified again using the DLS technique. The results of the analysis are shown in Figure 6.

The results obtained showed the effectiveness of the sonication process in the crushing of the magnetic nanoparticle agglomerates. The use of high-frequency sound waves resulted in the breaking up of the micrometric agglomerates of the particles. As a result, the suspension subjected to the sonication process for 4 h contained only particles with nanometric sizes. Thus, it could be concluded that this process was undoubtedly effective. Nonetheless, its disadvantage was the fact that the desired particle size (i.e., 1–100 nm) was achieved only after several hours. This, in turn, from a technological and ecological viewpoint, is uneconomical and time consuming. Therefore, in the next part of the research, two other reaction environments were proposed and investigated in terms of the colloidal stability of the particle suspension obtained, as well as the susceptibility of their agglomerates to the sonication process.

### 3.2. Studies on the Impact of the Reaction Environment on the Colloidal Stability of the Fe_3_O_4_@Ag Particle Suspension

The next steps included the synthesis of Fe_3_O_4_@Ag nanoparticles using an essential oil as a component of the reaction medium; the following variations were used:

- mixture of a 3% aqueous solution of Arabic gum and essential oil in a 1.4:1.0 volume ratio (oil/water (**O/W**) reaction environment);
- a 3% solution of Arabic gum in essential oil (oil (**O**) reaction environment).

The rest of the synthesis conditions (temperature, inert gas atmosphere, etc.), as well as the reagents used, were analogous to those in the case of the reaction performed in an aqueous reaction environment.

**Figure 7:**
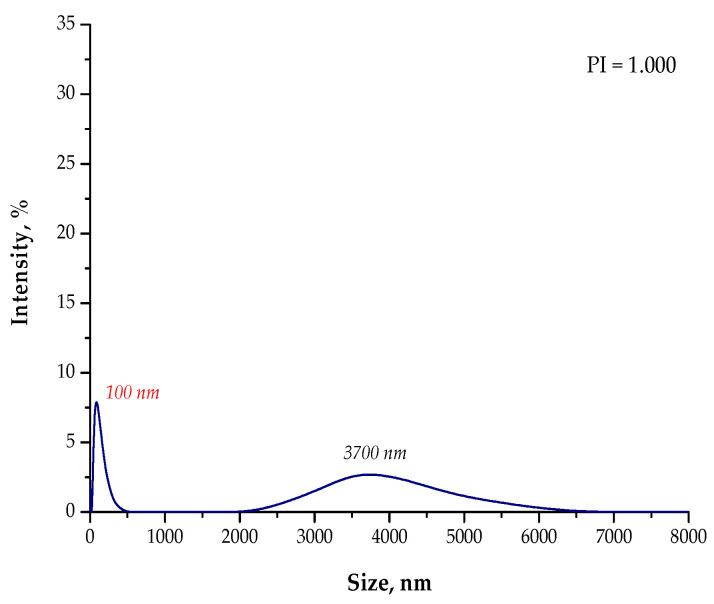
The size analysis of the Fe_3_O_4_@Ag particles obtained as a result of the synthesis performed using the 3% Arabic gum solution and the **O/W** reaction environment.

**Figure 8:**
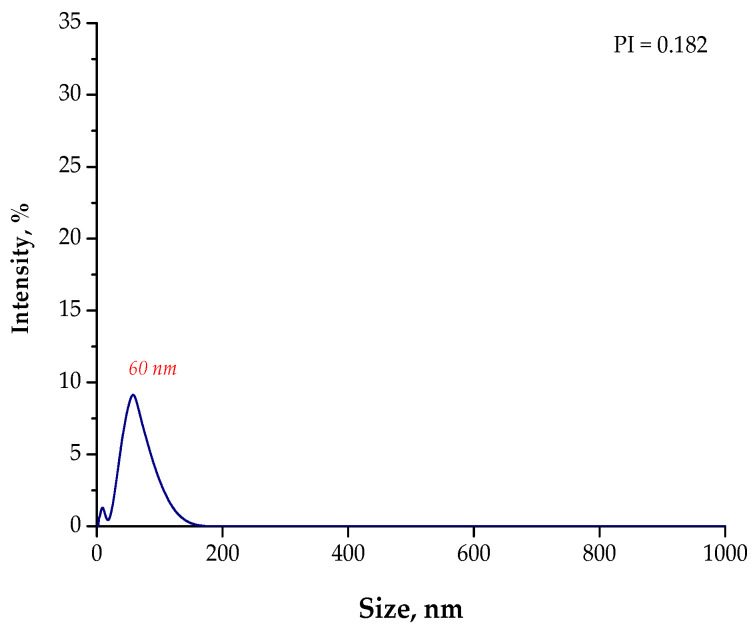
The size analysis of the Fe_3_O_4_@Ag particles obtained as a result of the synthesis performed using the 3% Arabic gum solution and the oil/water (**O/W**) reaction environment after 15 min of sonication.

**Figure 9:**
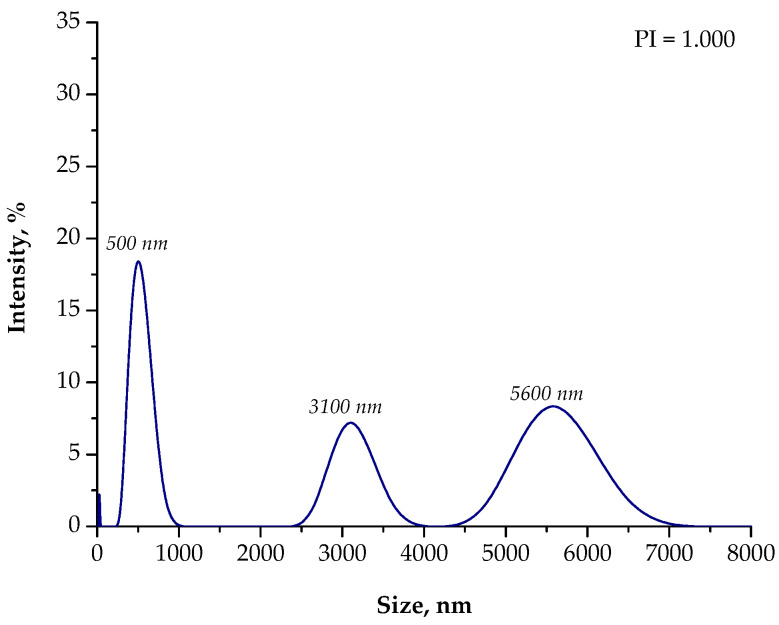
The size analysis of the Fe_3_O_4_@Ag particles obtained as a result of the synthesis performed using the 3% Arabic gum solution and the oil (**O**) reaction environment.

**Figure 10:**
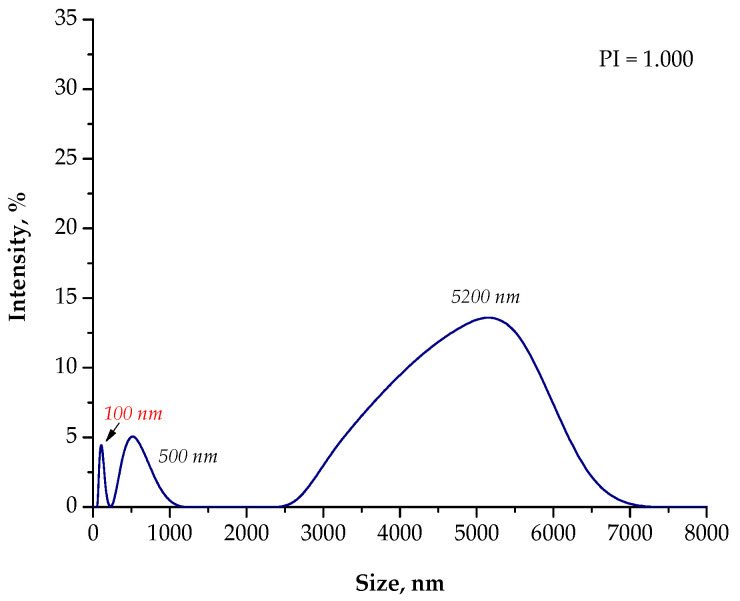
The size analysis of the Fe_3_O_4_@Ag particles obtained as a result of the synthesis performed using the 3% Arabic gum solution and the oil (**O**) reaction environment after 15 min of sonication.

In Figure 7, Figure 8, Figure 9 and Figure 10, the results of the size analysis of the particles obtained in the above-mentioned reaction environments are presented. Importantly, the measurements were performed both directly after the synthesis and after 15 min of sonication.

Based on the DLS analysis, it could be concluded that the introduction of an essential oil into the reaction medium influenced both the particle size distribution and the efficiency of the sonication process. In the case of the reaction performed using the oil/water reaction environment, the post-reaction suspension contained both microparticles with a size of approximately 3700 nm and nanoparticles. Thus, the sonication process was performed to break up the agglomerates. Importantly, after 15 min of subjecting the particle suspension to treatment with high-frequency sound waves, the resulting agglomerates were crushed. Only nanoparticles with a size of 60 nm were found in the tested suspension. Thus, it may be concluded that the oil/water reaction medium limited the formation of agglomerates compared to the water reaction environment (DLS analysis in Figure 4). In the case of the particle suspension obtained as a result of the synthesis performed in the water reaction medium, a much higher particle size distribution was observed. Moreover, the reaction performed using the 3% aqueous Arabic gum solution (without the essential oil) led to the preparation of particle agglomerates of various sizes, of which the largest were over 7000 nm in size. It was also demonstrated that the use of essential oil also affected the efficiency of the sonication process in the crushing of the particle agglomerates. This probably resulted from its chemical composition. It is an oily liquid containing numerous organic compounds, including aldehydes, ketones, esters, and terpenes, which are both aliphatic and aromatic. In the case of an oil/water reaction medium, the formation of an O/W emulsion was observed. The oil droplets were dispersed in the aqueous medium and, to some extent, limited the migration of nanoparticles. Thus, a decrease in the intensity of the interaction between them was observed, and as a consequence, the formation of particle agglomerates was strongly limited. Agglomerates were also formed (as in the case of the reaction performed without the essential oil as a component of the reaction medium), but with clearly smaller sizes; therefore, their breaking up occurred faster. The dispersed oil droplets caused additional depleted stabilization.

Another situation took place when using only essential oil as a reaction medium. Under these conditions, particles with a high polydispersity were obtained. In the tested suspension, particles with a size of 5600 nm (with an intensity of approximately 8.0%), 3100 nm (intensity of approximately 7.5%), and 500 nm (intensity of approximately 18.0%) were observed. Moreover, the presence of nanoparticles with a size of approximately 30 nm was also found. Thus, it may be concluded that particle agglomerates also occurred in the post-reaction suspension obtained as a result of the process performed in an oil reaction medium. Nonetheless, the agglomerates that formed were smaller than the agglomerates obtained as a result of the synthesis conducted using the water reaction environment.

The sonication of the particle suspension for 15 min did not provide the expected results. Agglomerates of a micrometric size were still present in the analyzed medium. In the case of the use of only essential oil as a reaction medium (i.e., a 3% solution of Arabic gum in essential oil), intense interactions between the Arabic gum and the chemical compounds present in the oil probably took place. This, in turn, limited the adsorption of this biopolymer onto the surface of the nanoparticles and, thus, limited the influence of steric stabilization. On the other hand, the depleted stabilization caused by the presence of Arabic gum and essential oil compounds turned out to be insufficient. This was due to the fact that, in the case of the oil reaction medium, the volume of the reaction mixture was much smaller than that in the case of the oil/water reaction medium. This, in turn, resulted in shorter distances between the nanoparticles and, thus, facilitated their interactions and the formation of agglomerates.

Based on the above-presented discussion, it may be concluded that the oil/water reaction medium is a medium conducive to the formation of nanoparticles. In such reaction conditions, the formation of particle agglomerates is strongly limited; after 15 min of sonication, a suspension containing only nanosized particles was obtained.

### 3.3. Characterization of the Chemical Structure of the Particles via FT-IR Spectroscopy

**Figure 11:**
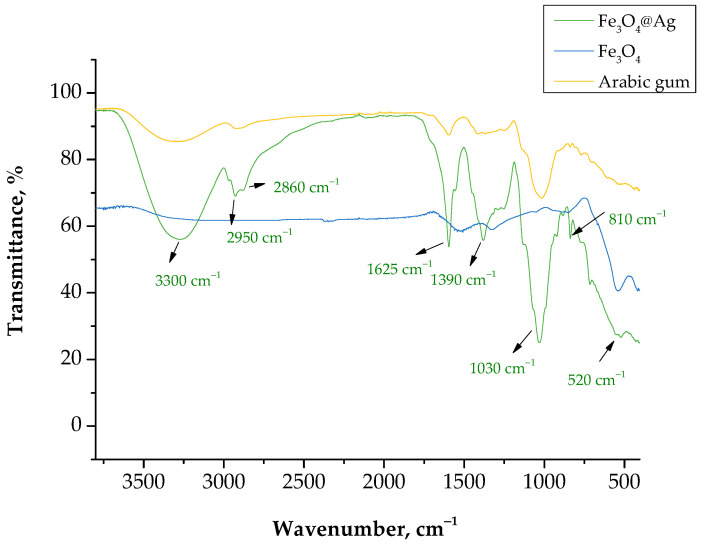
FT-IR spectrum of the Fe_3_O_4_@Ag nanoparticles (green line), Fe_3_O_4_ nanoparticles (blue line), and Arabic gum (orange line).

In Figure 11, the FT-IR spectrum of the Fe_3_O_4_@Ag nanoparticles obtained as a result of the reaction performed in the oil/water reaction medium is presented. An FT-IR analysis was also performed for Fe_3_O_4_ nanoparticles and Arabic gum.

FT-IR spectroscopy allowed the verification of the presence of specific functional groups in the structure of the developed materials. The absorption band at approximately 520 cm^−1^ corresponded to the tensile vibrations of Fe-O bonds derived from the magnetic core based on Fe_3_O_4_ nanoparticles [27]. Moreover, a number of absorption bands that probably came from the chemical bonds present in the structure of the compounds in the stabilizing agent used, i.e., Arabic gum, could be observed in the FT-IR spectrum. Arabic gum is a mixture of saccharides, such as galactose, arabinose or rhamnose, and glucuronic acid [28]. Thus, the bands at 2950 and 2860 cm^−1^ probably came from the stretching vibrations of the C-H bond, which is present in the structure of the saccharides (arabinose and rhamnose) and carboxylic acids in Arabic gum [29,30]. The intense band at 3300 cm^−1^ corresponded to the stretching vibrations of the –OH group [31]. It could also have come from the polysaccharides that are components of Arabic gum [32], as well as from the hydrogen bond formed between the oxygen atom in the Fe_3_O_4_ nanoparticles and the hydrogen from the hydroxyl group of the polysaccharides [33]. The absorption band at 1625 cm^−1^ probably came from the stretching vibrations of the –C=O bond, which is characteristic for saccharides or carboxylic acids [34]. Thus, it could be concluded that all absorption bands observed in the obtained FT-IR spectrum came from Fe_3_O_4_ nanoparticles or from the stabilizer used. This confirmed the previous conclusions on the stabilizing effect of Arabic gum, which was adsorbed onto the surface of nanoparticles, as a result of which steric stabilization occurred, limiting their interactions that would lead to the formation of nanoparticle agglomerates.

### 3.4. Characterization of the Particle Crystallinity via the XRD Technique

**Figure 12:**
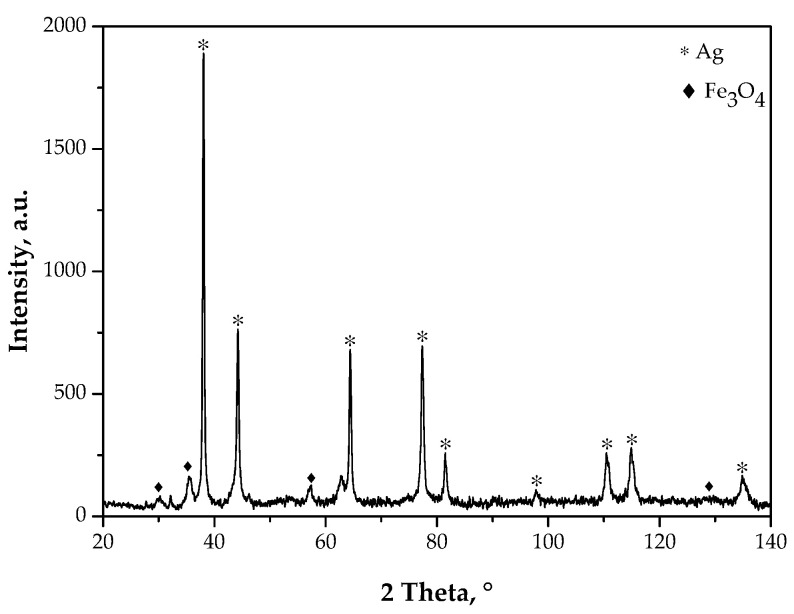
XRD diffractogram of the obtained Fe_3_O_4_@Ag nanoparticles.

The XRD patterns of the Fe_3_O_4_@Ag nanoparticles are presented in Figure 12.

In Table 1, the information obtained as a result of the XRD analysis is presented.

Based on the XRD diffractogram presented in Figure 12. It may be reported that the particles obtained had a crystalline structure. Clear XRD peaks were visible. Importantly, no background noise or amorphous halos were observed. 

The XRD diffraction patterns showed reflections at 2θ equal to 30.05°, 35.84°, and 57.85°. These peaks are characteristic for Fe_3_O_4_ (iron oxide) nanoparticle crystals with a cubic structure of an inverted spinel (Fe_3_O_4_, ICDD 00-001-1111) [35]. Furthermore, the presence of XRD peaks at 2θ equal to 38.75°, 44.20°, 64.50°, and 77.31° could be noticed. These peaks are characteristic of the regular face-centered cubic (fcc) structure of silver crystals (ICSS 00-004-0783) [36]. This, in turn, confirmed the presence of a nanosilver shell on the surface of the iron oxide nanoparticles. No reflections from other structures were reported, which proved the phase purity of the analyzed material.

### 3.5. Analysis of the Particles’ Surface Morphology Supported by an Elemental Composition Analysis via the SEM-EDS Method

**Figure 13:**
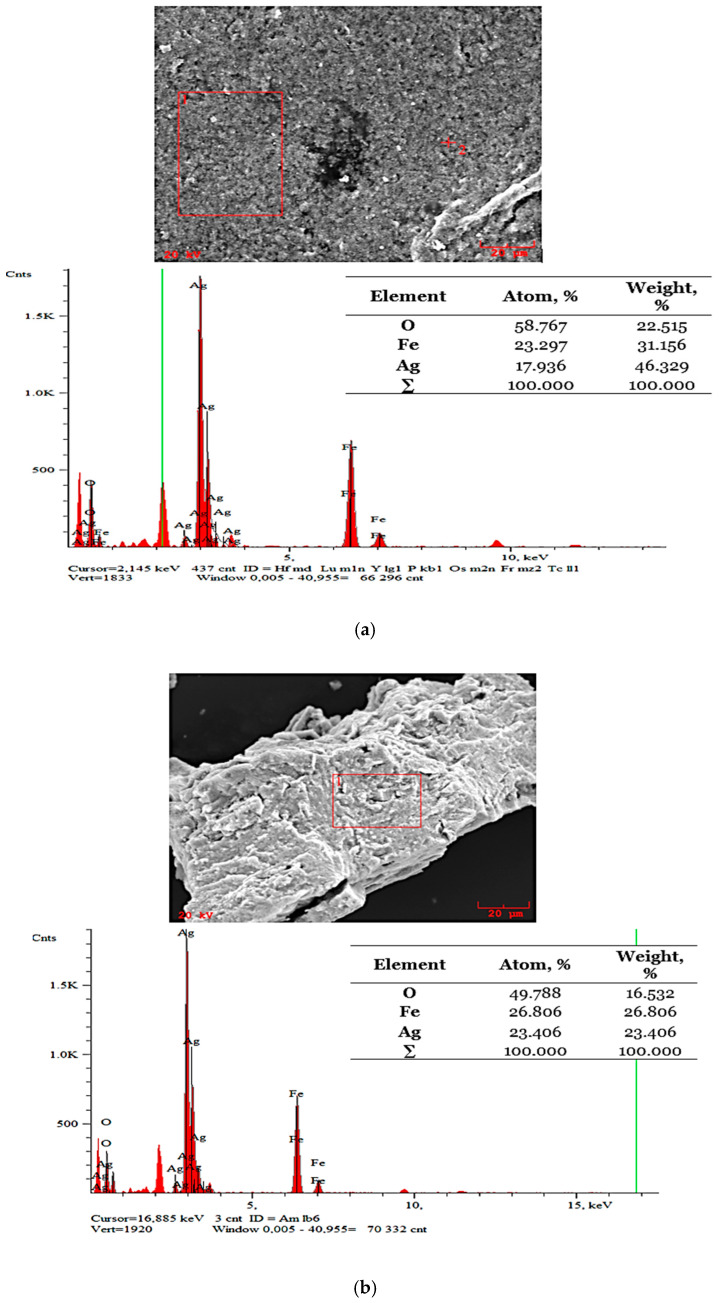
SEM images of the Fe_3_O_4_@Ag nanoparticles supported by an elemental analysis in two points (**a**,**b**).

The results of the SEM-EDS analysis performed on the Fe_3_O_4_@Ag nanoparticles are presented in Figure 13.

The EDS analysis allowed us to confirm the presence of iron, oxygen, and silver in the tested materials. Thus, it may be concluded that, as a result of the synthesis, Fe_3_O_4_@Ag nanoparticles were developed.

### 3.6. Analysis of the Particle Morphology via the TEM Technique

**Figure 14:**
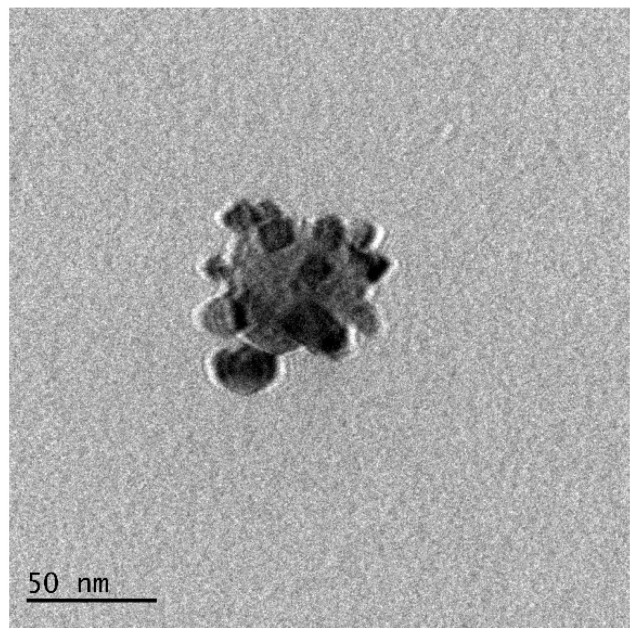
TEM image of the Fe_3_O_4_@Ag nanoparticles.

In Figure 14, a TEM image of the Fe_3_O_4_@Ag particles is presented.

In Figure 14, an iron oxide nanoparticle coated with smaller nanosized particles may be observed. Thus, the formation of core–shell structures was confirmed by this analysis.

### 3.7. Biological Investigations

#### 3.7.1. Results of the MTT Reduction Assay

**Figure 15:**
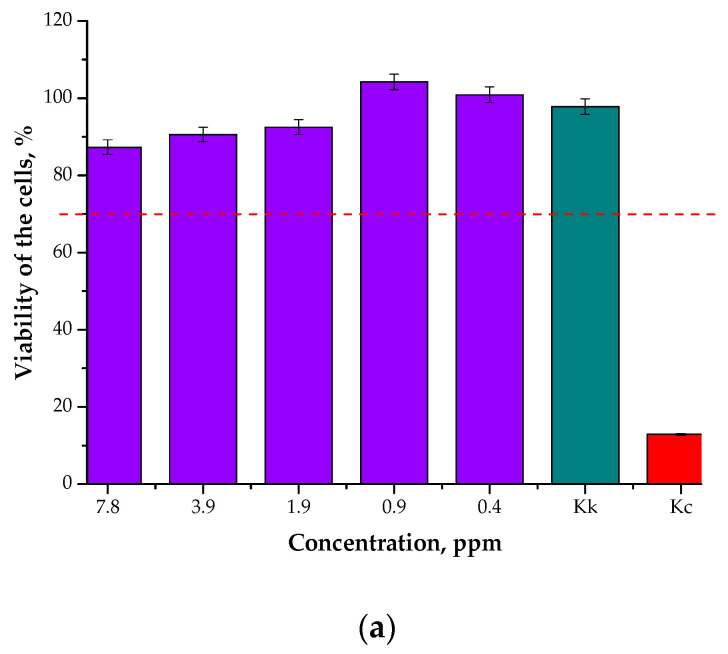

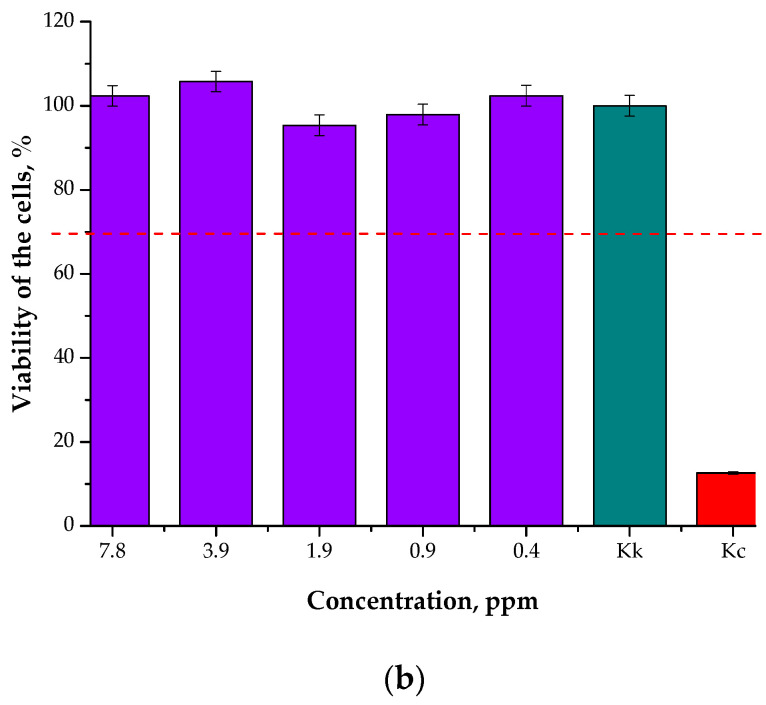
Results of the MTT reduction assay performed for the particle suspensions obtained in the water reaction medium (**a**) and the oil/water reaction medium (**b**).

In Figure 15, the results of the analysis of the cytotoxicity towards L929 murine fibroblasts are presented. The study was performed for both the particle suspension obtained through the synthesis in the water reaction medium and that obtained in the oil/water reaction medium to verify the impact of the reaction environment on the biological properties of the particles. As reference samples, cells treated with a well-known cytotoxic reagent, i.e., 1% phenol solution (Kc), and cells in a culture medium (Kk) were also investigated.

According to the relevant ISO standard, a material is determined to be non-cytotoxic when the survival rate of cells incubated in its presence for 24 h is over 70% [37]. Therefore, based on the analysis performed here, it may be concluded that the Fe_3_O_4_@Ag nanoparticle suspensions developed at the tested concentrations showed no cytotoxicity towards L929 murine fibroblasts. Thus, it may be reported that the prepared materials may be considered useful for use as drug carriers.

#### 3.7.2. Studies on the Pro-Inflammatory Activity of the Particles

**Figure 16:**
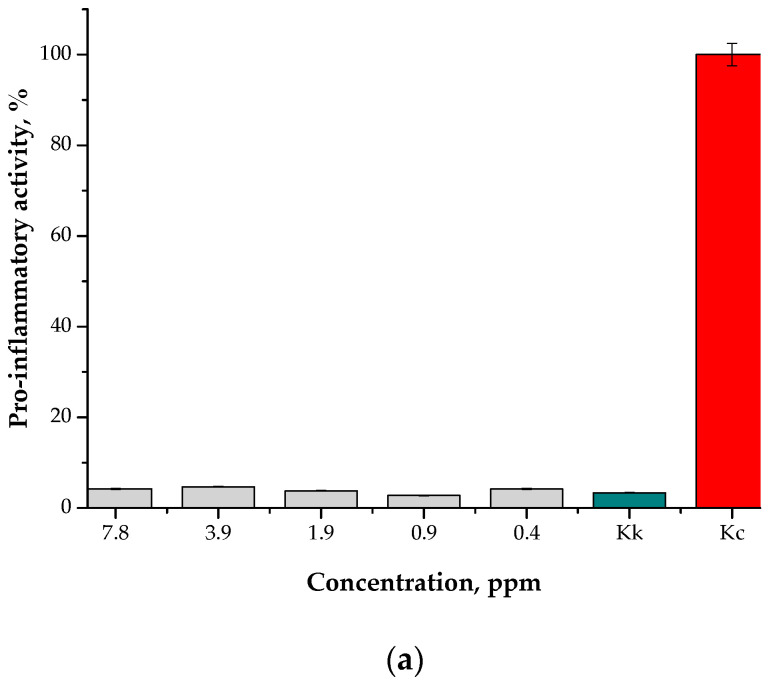

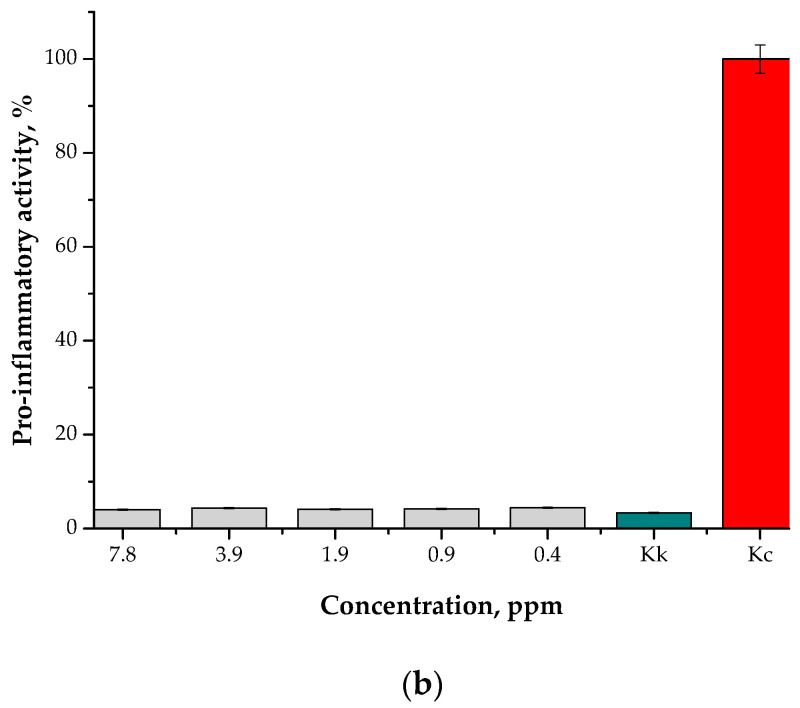
Results of the analysis of the pro-inflammatory activity of the particle suspensions obtained in the water reaction medium (**a**) and the oil/water reaction medium (**b**).

In Figure 16, the results of the pro-inflammatory studies are presented. As in the case of the MTT reduction assay, the study was conducted for the particle suspensions obtained in various reaction media to verify whether the reaction environment affected the pro-inflammatory activity of the resulting materials. As reference samples, cells treated with a reagent showing pro-inflammatory activity (Kc) and cells in a culture medium (Kk) were also tested.

The results of this study allowed us to state that the nanoparticle suspensions (at all tested concentrations), regardless of the reaction environment, showed no pro-inflammatory activity. The results for the tested suspensions were similar to the results for unstimulated monocytes in the culture medium (Kk control).

## 4. Conclusions

- The synthesis performed here allowed us to obtain magnetic nanoparticles with a shell made of silver nanoparticles. This shell was confirmed via UV-Vis spectrophotometry, X-ray diffraction (XRD peaks characteristic for the regular face-centered cubic (fcc) structure of silver crystals (ICSS 00-004-0783)), and the elemental composition.
- It was demonstrated that the stabilizer concentration affected the size of the particles and the susceptibility of agglomerates thereof to crushing. Arabic gum in the form of a 3% aqueous solution was an efficient stabilizing agent.
- It was proven that the oil/water reaction medium was conducive to the formation of nanoparticles. In these reaction conditions, the formation of particle agglomerates was strongly limited; after 15 min of sonication, a suspension containing only nanosized particles was obtained.
- Regardless of the reaction environment, the obtained nanomaterials exhibited no cytotoxic properties towards L929 murine fibroblast cells or pro-inflammatory activity against the THP1XBlueTM cell line.
- The possibility of the modification of magnetic nanoparticles makes them promising for application as drug carriers. Additionally, due to their magnetic properties, an active substance can be delivered to a specific site via an external magnetic field.

## Figures and Tables

**Table 1:** Parameters determined based on the XRD diffraction patterns of the Fe_3_O_4_@Ag nanoparticles.

Chemical Compound (ICDD)	Space Group	Network Type	Pattern Network Parameter (ICDD), Å	Calculated Network Parameter, Å	Network Deformation, %	Crystallinity Size, nm
Fe_3_O_4_ (00-001-1111)	Fd-3m	cubic	a = 8.374	a = 8.371	0	10
Ag (00-004-0783)	Fm-3m	cubic	a = 4.0862	a = 4.0857	−0.10	60

## Data Availability

Data sharing is not applicable for this article.
